# Supplementary material for: An epigenetic switch ensures transposon repression upon dynamic loss of DNA methylation in embryonic stem cells
Source: eLife. 2016 Jan 27;5:e11418. doi: 10.7554/eLife.11418 (PMC4769179; doi:10.7554/eLife.11418)
Supplement: Supplementary file 2. — Elements were considered as 'active' when they were covered by at least 10 uniquely mapped reads at one of the time point. Percentages represent the proportion of active copies relative to the total number of elements in a given family, as estimated from the reconstructed version of RepeatMasker. For ERVs, solo-LTRs were excluded and numbers represent only elements containing internal sequences. (B) Gene Ontology enrichment analysis for genes specifically up-regulated between D0 and D6. Gene ontology analysis for biological processes and molecular functions was performed for the 156 genes that were significantly up-regulated between D0 and D6 but with no significant differences between D0 and D13. The ten most significant terms are shown. (C) List of chimeric transcripts identified during conversion from serum to 2i+vitC. The 30 genes with the highest number of chimeric reads at D6 are ranked here. Numbers represent the absolute read count at the junction between the transposon (first exon) and the second exon of the gene, and the normalized read count of the whole transcript in RPKM. (D) Primer and sequence list. (E) Antibody list. DOI: http://dx.doi.org/10.7554/eLife.11418.026 [file elife-11418-supp2.docx]

|  | number (and percentage) of unique elements expressed at | | | |  | total number of elements |
| --- | --- | --- | --- | --- | --- | --- |
|  | **D0** | **D6** | **D13** | **throughout conversion** |  |  |
| All LINE1s | 4,334 (0.7%) | 5,659 (1%) | 2,303 (0.4%) | 7,163 (1.2%) |  | 588,739 |
| All ERVs | 1,229 (2.0%) | 1,925 (3.1%) | 1,381 (2.2%) | 2,372 (3.8%) |  | 62,098 |
| L1Md_A | 1,068 (8.1%) | 1,620 (12.3%) | 425 (3.2%) | 1,891 (14.4%) |  | 13,172 |
| L1Md_T | 1,631 (9.2%) | 2,129 (12.0%) | 438 (2.5%) | 2,447 (13.8%) |  | 17,740 |
| IAPEz | 45 (1.5%) | 127 (4.3%) | 63 (2.1%) | 174 (5.9%) |  | 2,958 |
| IAPEy | 9 (1.8%) | 66 (12.9%) | 64 (12.5%) | 85 (16.6%) |  | 512 |
| ETnERV | 119 (6%) | 159 (8.1%) | 94 (4.8%) | 178 (9.0%) |  | 1,969 |
| MERVL | 118 (3.4%) | 110 (3.2%) | 56 (1.6%) | 159 (4.6%) |  | 3,444 |
| VL30 | 4 (1.2%) | 12 (3.5%) | 3 (0.9%) | 13 (3.8%) |  | 343 |
| MMERGLN | 7 (2.3%) | 68 (22,6%) | 53 (17,6%) | 71 (23,6%) |  | 300 |
| malR-ORR1A | 210 (3.9%) | 349 (6.6%) | 265 (5.0%) | 447 (8.5%) |  | 5,256 |

**Supplementary file 2A - Number and percentage of active transposable elements at D0, D6 and D13 during conversion from serum to 2i+vitC.** Elements were considered as “active” when they were covered by at least 10 uniquely mapped reads at one of the time point. Percentages represent the proportion of active copies relative to the total number of elements in a given family, as estimated from the reconstructed version of RepeatMasker. For ERVs, solo-LTRs were excluded and numbers represent only elements containing internal sequences.

| Biological Process | P-value |
| --- | --- |
| multicellular organismal development (GO:0007275) | 2.890e-05 |
| single-multicellular organism process (GO:0044707) | 1.004e-04 |
| multicellular organismal process (GO:0032501) | 1.875e-04 |
| anatomical structure development (GO:0048856) | 2.711e-04 |
| single-organism developmental process (GO:0044767) | 2.929e-04 |
| developmental process (GO:0032502) | 3.279e-04 |
| anatomical structure morphogenesis (GO:0009653) | 3.383e-04 |
| system development (GO:0048731) | 5.315e-04 |
| organ development (GO:0048513) | 1.275e-03 |
| single-organism process (GO:0044699) | 3.169e-03 |

| Molecular Function | P-value |
| --- | --- |
| nucleic acid binding transcription factor activity (GO:0001071) | 9.731e-03 |
| sequence-specific DNA binding transcription factor activity (GO:0003700) | 9.731e-03 |
| regulatory region nucleic acid binding (GO:0001067) | 5.069e-02 |
| regulatory region DNA binding (GO:0000975) | 5.069e-02 |
| transcription regulatory region DNA binding (GO:0044212) | 5.069e-02 |
| sequence-specific DNA binding RNA polymerase II transcription factor activity (GO:0000981) | 8.025e-02 |
| molecular_function (GO:0003674) | 1.229e-01 |
| RNA polymerase II core promoter proximal region sequence-specific DNA binding transcription factor activity (GO:0000982) | 2.048e-01 |
| RNA polymerase II transcription regulatory region sequence-specific DNA binding transcription factor activity involved in negative regulation of transcription (GO:0001227) | 2.774e-01 |
| DNA binding (GO:0003677) | 6.186e-01 |

**Supplementary file 2B – Gene Ontology enrichment analysis for genes specifically up-regulated between D0 and D6.** Gene ontology analysis for biological processes and molecular functions was performed for the 156 genes that were significantly up-regulated between D0 and D6 but with no significant differences between D0 and D13. The ten most significant terms are shown.

|  |  | number of reads at the junction transposon-2nd exon | | | RPKM | | |
| --- | --- | --- | --- | --- | --- | --- | --- |
| Gene | **Transposon** | **D0** | **D6** | **D13** | **D0** | **D6** | **D13** |
| Prrc1 | RLTR16B_MM | 542 | 4202 | 4583 | 5.1 | 49.7 | 70.7 |
| Bglap3 | IAP-d-int | 29 | 2420 | 3061 | 0.1 | 5.7 | 4.1 |
| Gab1 | RLTR15 | 2823 | 1714 | 1261 | 142.5 | 113.1 | 100.0 |
| Mylpf | ID_B1 | 537 | 1691 | 1093 | 28.1 | 143.3 | 116.3 |
| Pecam1 | RLTR11B | 869 | 1493 | 1530 | 20.2 | 36.5 | 33.8 |
| Usp7 | ORR1A4 | 994 | 1360 | 1077 | 40.7 | 55.2 | 48.0 |
| Plcb4 | RLTR17 | 209 | 1093 | 716 | 5.7 | 4.2 | 2.8 |
| Cyp2b23 | ERVB7_2B-LTR_MM | 16 | 860 | 356 | 0.2 | 4.2 | 3.3 |
| Nfu1 | Lx7 | 785 | 637 | 468 | 5.8 | 4.6 | 3.7 |
| Mep1b | RLTR9E | 66 | 625 | 159 | 0.3 | 3.1 | 0.6 |
| Dopey2 | Lx7 | 74 | 591 | 258 | 2.2 | 16.1 | 11.0 |
| 1500012F01Rik | B2_Mm2 | 571 | 534 | 198 | 20.7 | 20.5 | 8.1 |
| Cdyl2 | RLTR15 | 230 | 496 | 482 | 5.1 | 13.6 | 12.9 |
| Serpina3m | MMERVK9C_I-int | 2 | 488 | 507 | 0.0 | 6.9 | 6.6 |
| Wdr95 | L1MB2 | 24 | 443 | 433 | 0.2 | 5.1 | 5.2 |
| 4930548H24Rik | BGLII | 88 | 419 | 385 | 0.6 | 4.1 | 4.2 |
| Kdm2b | B4A | 14 | 401 | 158 | 1.1 | 26.5 | 16.6 |
| Ppm1a | MT2B | 87 | 393 | 265 | 0.8 | 2.6 | 1.9 |
| Atg4b | RLTR12D | 60 | 358 | 311 | 2.1 | 9.6 | 8.0 |
| Pla2g1b | RLTR11A | 874 | 357 | 155 | 21.2 | 6.5 | 3.9 |
| Fbrsl1 | RLTR11B | 324 | 325 | 168 | 12.7 | 13.7 | 10.8 |
| Hcrtr2 | ORR1B2 | 47 | 307 | 220 | 0.3 | 1.2 | 0.4 |
| Xpot | RMER6B | 250 | 297 | 132 | 5.9 | 7.0 | 4.0 |
| Sec24d | ORR1D1 | 424 | 276 | 127 | 2.0 | 2.9 | 0.5 |
| Oas1f | MTD-int | 4 | 257 | 544 | 0.1 | 9.7 | 21.7 |
| Atg13 | L1MB3 | 155 | 243 | 57 | 3.8 | 7.2 | 2.1 |
| Cul5 | RLTR20C1 | 98 | 236 | 125 | 4.5 | 7.3 | 8.0 |
| Mybl2 | B1_Mur3 | 224 | 223 | 121 | 9.2 | 9.4 | 6.3 |
| Aoah | BLII_Mus | 101 | 212 | 89 | 5.1 | 10.6 | 2.8 |
| Ccbl2 | RLTR44-int | 2 | 201 | 148 | 0.0 | 1.7 | 1.6 |

**Supplementary file 2C – List of chimeric transcripts identified during conversion from serum to 2i+vitC.** The 30 genes with the highest number of chimeric reads at D6 are ranked here. Numbers represent the absolute read count at the junction between the transposon (first exon) and the second exon of the gene, and the normalized read count of the whole transcript in RPKM.

| RT-qPCR and ChIP-qPCR |  |
| --- | --- |
| RplP0 F | TCCAGAGGCACCATTGAAATT |
| RplP0 R | TCGCTGGCTCCCACCTT |
| Gapdh F | TCCATGACAACTTTGGCATTG |
| Gapdh R | CAGTCTTCTGGGTGGCAGTGA |
| LINE1 F (ORF2) | GGAGGGACATTTCATTCTCATCA |
| LINE1 R (ORF2) | GCTGCTCTTGTATTTGGAGCATAGA |
| IAPEz F (IAPdelta1 subfamily) | AACGCTGCTGCTTTAACTCC |
| IAPEz R (IAPdelta1 subfamily) | TGCACATAAAGCTGGCACA |
| MERVL F | CAATGGGAAGGTCCAGAAGA |
| MERVL R | CCTTGTTACCTCGGAATCCA |
| L1-T F (T monomer) | CAGCGGTCGCCATCTTG |
| L1-T R (T monomer) | CACCCTCTCACCTGTTCAGACTAA |
| L1-A F (A monomer) | GGATTCCACACGTGATCCTAA |
| L1-A R (A monomer) | TCCTCTATGAGCAGACCTGGA |
| IAPEz 5 ‘UTR F (for ChIP) | CGGGTCGCGGTAATAAAGGT |
| IAPEz 5 ‘UTR F (for ChIP) | ACTCTCGTTCCCCAGCTGAA |
| Major Satellite F | GACGACTTGAAAAATGACGAAATC |
| Major Satellite R | CATATTCCAGGTCCTTCAGTGTGC |

| CRISPR guide sequence (without PAM) | |
| --- | --- |
| Trim28 exon 3 5' | GCAAGTAAATACAGGTCTGC |
| Trim28 exon 3 3' | GCAGACTTTGGAGGTTTAGG |
| Suvar39h1 exon 4 | GGCCAGATCTACGACCGCCA |
| Suvar39h2 exon 4 5' | TCTTCACTTGTGATCACCTA |
| Suvar39h2 exon 4 3' | ACAGTGGATGCAGCTCGATA |
| Setdb1 exon 16 | AGATGGCAACAGCGGTTCAG |
| Eed exon 6 5’ | TCCCTTAGTTCAGTTTGTTT |
| Eed exon 6 3’ | CCTTGAGTGTACTAGGCTAT |

| Bisulfite pyrosequencing | |  |
| --- | --- | --- |
| Dazl F | TTTAGGATTTATTTTATAGGGGT | |
| Dazl R [Btn] | CAAAAAAAACCAAAAAACCCA | |
| Dazl Seq | GGGGGGTTAGGGAGTG | |
| H19 F | GGGTTTTTTTGGTTATTGAATTTTAA | |
| H19 R [Btn] | AATACACACATCTTACCACCCCTATA | |
| H19 Seq | TGTTATGTGTAATAAGGGAA | |
| Oct4 F | AGGGGTGAGAGGATTTTGAA | |
| Oct4 R [Btn] | ACCTCTCCCTCCCCAATC | |
| Oct4 Seq | GGTTGAAAATGAAGGTTT | |
| IAP F | GAGGGTGGTTTTTTATTTTATGTGT | |
| IAP R [Btn] | ATCACTCCCTAATTAACTACAACC | |
| IAP Seq | TTTTTATTTTATGTGTTTTGTTTTT | |
| L1-T F | GGTTGGGGAGGAGGTTTAAGTTATA | |
| L1-T R [Btn] | CTACCTATTCCAAAAACTATCAAATTCTT | |
| L1-T Seq | GGGAGGAGGTTTAAGTTATAGTA | |
| L1-A F | AGATTGAGGTATATAGGGAAGTAGGTT | |
| L1-A R [Btn] | ATCCACTCACCAAAAATCTTAAAAT | |
| L1-A Seq | GGTATATAGGGAAGTAGGTTA | |

| Nanostring nCounter target sequences | |  |
| --- | --- | --- |
| Ppia | CCAAGACTGAATGGCTGGATGGCAAGCATGTGGTCTTTGGGAAGGTGAAAGAAGGCATGAACATTGTGGAAGCCATGGAGCGTTTTGGGTCCAGGAATGG | |
| Gapdh | AGGTTGTCTCCTGCGACTTCAACAGCAACTCCCACTCTTCCACCTTCGATGCCGGGGCTGGCATTGCTCTCAATGACAACTTTGTCAAGCTCATTTCCTG | |
| RplP0 | AAGGAAGAGTCGGAGGAATCAGATGAGGATATGGGATTCGGTCTCTTCGACTAATCCCGCCAAAGCAACCAAGTCAGCCTGCTTAATTTGAGAAAGATGG | |
| Actb | AGTTCGCCATGGATGACGATATCGCTGCGCTGGTCGTCGACAACGGCTCCGGCATGTGCAAAGCCGGCTTCGCGGGCGACGATGCTCCCCGGGCTGTATT | |
| PCNA | AGACCTTAGCCACATTGGAGATGCTGTTGTGATATCCTGTGCAAAGAATGGGGTGAAGTTTTCTGCAAGTGGAGAGCTTGGCAATGGGAACATTAAGTTG | |
| Ki-67 | TGATTTGCTCCAAAAGGCGAAGTGGAGCTTCTGAAGCCAACTTGATTGTTGCAAAATCATGGGCTGATGTTGTAAAACTTGGCGTGAAACAAACACAAAC | |
| Top2a | TACTACAAAGGTTTGGGCACCAGCACATCAAAGGAAGCTAAGGAATATTTTGCAGATATGAAACGACATCGTATTCAGTTCAAATACTCTGGCCCTGAAG | |
| IAPEz | TTGACTTGTTAACGGGTCAGAGAGCTTATTCTGCTAAACCTGATAAGAGGTATCAATGGAAGGTCTTACCACAGGGGATGTCCAATAGTCCTACAATGTG | |
| Line1 ORF2 | AATGGCTAAGATCAAAAATTCAGGTGACAGCAGATGCTGGCGAGGATGTGGAGAAAGAGGAACACTCCTCCATTGTTGGTGGGAGTGCAGGCTTGTACAA | |
| L1-A | GGCTACCCGGGCCTGATCTGGGGCACAAGTCCCTTCCGCTCGACTCGAGACTCGAGCCCCGGGCTACCTTGCCAGCAGAGTCTTGCCCAACACCCGCAAG | |
| L1-T | CCGGCTGGGGAGGCGGCCTAAGCCACAGCAGCAGCGGTCGCCATCTTGGTCCGGGACCCGCCGAACTTAGGAAATTAGTCTGAACAGGTGAGAGGGTGCG | |
| MERVL | GTATTCCTGGAGAAATTGCAGAAATTACTGCCACTATCAAGGACTTGAAAGATGCAGGGGTGGTGGTTCCCACCACATCTCCGTTTAACTCTCCTATCTG | |
| VL30 | GCCTTCTAAAATAAGCCTAAAAATCCTGTCAGATCCCTATGCTGACCACTTCCTTTCAGATCAACAGCTGCCCTGCCTCCCACTCCAACTCCAGAGAGCA | |

**Supplementary file 2D – Primer and sequence list**

| Antigen target | Supplier/Reference | Usage (dilution) |
| --- | --- | --- |
| LINE1-ORF1 | gift from A. Bortvin | Western (1/1000) - IF (1/1000) |
| IAP-GAG | gift from B. Cullen | IF (1/200) |
| KAP1 | Abcam ab10483 | Western (1/1000) |
| PARP | Cell Signaling | IF (1/200) |
| H3S10phospho | Cell signaling 9706 | IF (1/500) |
| H3K9me2 | Abcam ab1220 | Western (1/1000) - IF (1/200) - ChIP (5μg) |
| H3K9me3 | Abcam ab8898 | Western (1/1000) - IF (1/200) - ChIP (3μg) |
| H3K27me3 | Cell Signaling C36B11 | Western (1/1000) - IF (1/200) - ChIP (5μL) |
| H3K4me3 | Milipore 07-473 | ChIP (3μL) |
| OCT4 | Abcam ab19857 | Western (1/1000) |
| NANOG | Abcam ab70482 | Western (1/1000) |
| KLF4 | Santa Cruz sc20691 | Western (1/1000) |
| SOX2 | Millipore AB5603 | Western (1/1000) |
| H3 | Abcam ab1791 | Western (1/5000) |
| TUBULIN | Millipore CP06 | Western (1/5000) |
| SUV39H1 | Cell signaling 8729S | Western (1/1000) |

**Supplementary file 2E – Antibody list**
